# Maximizing the Potential of Patient-Reported Assessments by Using the Open-Source Concerto Platform With Computerized Adaptive Testing and Machine Learning

**DOI:** 10.2196/20950

**Published:** 2020-10-29

**Authors:** Conrad Harrison, Bao Sheng Loe, Przemysław Lis, Chris Sidey-Gibbons

**Affiliations:** 1 Nuffield Department of Orthopaedics, Rheumatology and Musculoskeletal Sciences University of Oxford Oxford United Kingdom; 2 The Psychometrics Centre University of Cambridge Cambridge United Kingdom; 3 MD Anderson Center for INSPiRED Cancer Care University of Texas Houston, TX United States

**Keywords:** computerized adaptive testing, computerized adaptive test, CAT, machine learning, patient reported outcome measures, outcome assessment, Concerto

## Abstract

Patient-reported assessments are transforming many facets of health care, but there is scope to modernize their delivery. Contemporary assessment techniques like computerized adaptive testing (CAT) and machine learning can be applied to patient-reported assessments to reduce burden on both patients and health care professionals; improve test accuracy; and provide individualized, actionable feedback. The Concerto platform is a highly adaptable, secure, and easy-to-use console that can harness the power of CAT and machine learning for developing and administering advanced patient-reported assessments. This paper introduces readers to contemporary assessment techniques and the Concerto platform. It reviews advances in the field of patient-reported assessment that have been driven by the Concerto platform and explains how to create an advanced, adaptive assessment, for free, with minimal prior experience with CAT or programming.

## Introduction

### Patient-Reported Assessment 

Patient-reported outcome measures (PROMs) measure the outcomes of health care that are most meaningful to patients. The importance and validity of a patient-centered approach to outcome assessment is gaining widespread acceptance across a diverse range of stakeholders including clinicians, researchers, clinical commissioners, health care business strategists, and patients themselves [1-4]. 

Despite growing interest in the use of patient-reported assessments, there are considerable barriers to their use, especially in time-pressured clinical environments. Two barriers, in particular, are both problematic and addressable. First, questionnaires can be burdensome to complete, especially when multiple domains of patient health are assessed at the same time [5]. Second, it can be unclear to health care professionals what actions should be taken based on the information received. In this paper, we will discuss strategies to reduce the burden of patient-reported assessment and improve the actionability and relevance of feedback. Specifically, it introduces modern psychometric theories, which can be used to create individualized assessments and reduce the burden of completion, as well as techniques for providing individualized feedback.

### Modern Psychometrics and Item-Response Theory

The accuracy, reliability, and validity of patient-reported assessments are underpinned by complex psychometric statistical theory. Applying psychometric methods ensures that scores generated from PROMs can be used with high confidence in clinical practice and research [6].

Psychometrics can be divided into two broad domains. The first, referred to as classical test theory, uses correlational statistics to assess questionnaire properties, for example, how well the responses to certain items (questions) correlate with each other [7]. The second, known as modern test theory, uses probabilistic models to determine the properties of individual items [6].

The benefit of using modern test theory over classical test theory is that modern test theory allows researchers to evaluate the psychometric properties of individual items in relation to a targeted trait, whereas classical test theory correlational statistics mainly focuses on test-level performance. A more detailed comparison between the two approaches can be found elsewhere [6-8]. One of the biggest advantages of using modern test theory is that item properties can be used in a computerized adaptive testing (CAT) environment.

### CAT

CAT refers to a process of selecting the most informative items for people responding to questionnaires. In contrast to fixed-length assessments, where a standard set of items are presented to every respondent all at once, CAT employs a psychometric algorithm to select items one at a time based on the amount of information they will provide about the individual assessment taker [9]. Each item is calibrated using statistical models described by modern test theory, and this process provides parameters for each item which are then used by the CAT algorithm to both calculate a respondent’s score and select items [5].

After each response, CAT algorithms calculate a respondent’s score based on the information available and select the next most informative item to administer. As more items are answered, the person’s score is calculated with increasing accuracy. The CAT will eventually terminate when a *stopping rule* has been met. Stopping rules are typically based on a prespecified time limit, the number of items, the minimum standard error of measurement (SEM), or a combination thereof. Adapting assessments in this way can make them either briefer, more accurate, or in certain cases, both [10].

Many studies have assessed the impact of CAT on the length and accuracy of patient-reported outcome assessments. Experiments conducted both in silico and using human participants have robustly demonstrated that CAT can reduce the length of assessments by more than 50% while keeping excellent agreement between fixed-length assessment scores and CAT [5,11-14].

Despite their impressive performance, the uptake of currently available CAT platforms has been limited, including the Patient-Reported Outcome Measurement Information System (PROMIS) CAT which is accessible in the United States within the Epic electronic health record system [15]. In order to improve the uptake of this transformative technology and move toward truly patient-centered care, we must provide a more accessible way to implement CAT platforms in clinical practice and research. Research has demonstrated that PROM interventions are likely to have the greatest positive impact on patient outcomes when they are closely aligned with clinical care [16], and to achieve this we must administer such interventions through a versatile platform that meets the needs of clinicians, researchers, and patients. 

### Machine Learning

Machine learning refers to the process of developing or *training* algorithms to recognize patterns in existing data and to use this knowledge to make successful predictions with new data [17]. A great deal of enthusiasm has been shown for machine learning, as it has demonstrated exceptional performance in a variety of tasks including predicting the outcome of individuals following certain medical interventions, interpreting diagnostic images, and assessing the meaning of open-text passages [17-20].

There are a number of ways in which the collection and analysis of patient-reported data could be improved using machine learning. For instance, a branch of machine learning known as natural language processing may be used to generate quantifiable information from unstructured passages of open text [20]. Patient-reported assessments with integrated machine learning functions could include written (or spoken) patient responses, quantify said responses in a meaningful way, and use them to make recommendations for patient care or service improvement. 

### Concerto

Concerto was developed with the intention of providing a secure, versatile, and easy-to-use platform for creating patient-reported assessments that can incorporate CAT and machine learning. It is free to use and features a point-and-click interface that can be used to build advanced assessments with minimal prior programming experience [21].

Assessments created in Concerto are administered through highly adaptable front-end user interfaces that can be accessed from computers, smartphones, and electronic tablets. These interfaces are built similarly to websites, using HTML, JavaScript and CSS. The Concerto platform comes with inbuilt stock templates for users that do not wish to write their own code.

This user interface interacts with back-end functions, which can include scoring, CAT, and/or machine learning algorithms, using the R programming language. R programming has become popular among statisticians and data scientists for its breadth and accessibility [22]. There are currently over 15,000 available R packages, which can be used free of charge for statistical computing tasks including psychometric analyses, adaptive testing, and machine learning [23]. Concerto incorporates prewritten R code that can administer non-adaptive assessments or computerized adaptive tests, using item parameter tables that are uploaded by the user. The code is fully customizable for developers wishing to create more specialist assessments.

Patient data are stored securely using the MySQL database management system. The Concerto platform itself can be installed on Amazon cloud-based servers that comply with rigorous security demands. Alternatively, it can be installed on local servers (eg, those belonging to a health care provider) to comply with institutional security protocols. This has enabled the platform to be used successfully in clinical trials and for routine clinical care in the British National Health Service [24].

The Concerto platform can present assessment results immediately. Assessments can be presented in many forms depending on the needs of the end user. For example, radial plots can capture multiple dimensions of a person’s health state (eg, different PROM subscale scores) at discrete times, and trend plots can show how a person’s scores have changed over time or following major clinical events. Scores can be compared to normative values or other interpretability estimates, and SEMs can be presented alongside CAT scores. Respondents can even receive personalized written feedback to contextualize results (eg, “Your result is… This means…”). Providing immediate graphical and text-based feedback in this way has been shown to improve the experience of assessment when compared with traditional administration [2]. Results can be directly imported into a person’s electronic health record through application programming interfaces.

In the following section, we demonstrate how a new Concerto user can create a computerized adaptive test for the Centre for Epidemiologic Studies Depression (CES-D) PROM.

## Concerto: a Worked Example

### Installation

Up to date installation guidance for personal Concerto use can be found at the Concerto GitHub webpage [25]. Readers should be aware that if they choose to use the Amazon Web Service (AWS) for installation, they will need to submit credit or debit card details as part of the registration process. Provided the default instance type (t2.micro) is selected, the following exercise should fall under Amazon’s Free Tier. Readers are solely responsible for any costs they incur, and we would recommend that inexperienced AWS users take care when using the service.

### Download CES-D Items

To complete this exercise, readers will need to download a CSV file that contains the item wordings, item response theory parameters and response options for the CES-D. This is available to download from the Open Science Framework [26].

We will refer to this table as a “flat” item table because all the data are stored in one layer (ie, there are no sub-tables within it).

Download the flat item table, install Concerto, and log in.

### Create a New Assessment

On the *Tests* tab, click *Add new*.

Enter the name of your assessment in the *Name* box (eg, CESD_adaptive). The *Type* dropdown box should be set to *flowchart*. Click *Save*. Your test should appear under the *Tests* tab.

### Create a Table to Store Item Responses

**Figure 1:**
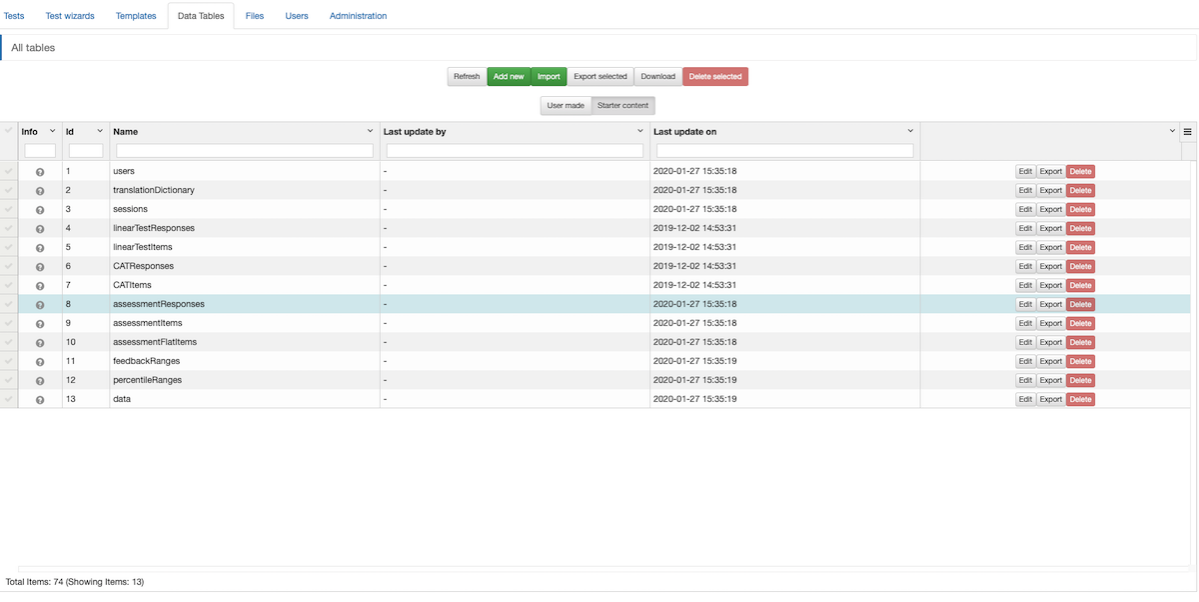
Concerto screenshot: creating a data table to store item responses.

Click on the *Data Tables* tab and select *Starter content*. Click *Edit* next to the *assessmentResponses* table (see Figure 1). Click *Copy* and change the name of the table (eg, CESDResponses). Click *Save*. This action saves a new table in which to store your responses.

### Upload Your Items

Click on *All tables* and select *User made*. Click *Add new* and type the name of your item table (eg, CESDFlatItems). Click *Save*. Your item table should appear in the list of user-made tables.

Click *Edit* next to your new item table. Click *Upload CSV*. Check the *Restructure* and *Header row* boxes. Use the *Choose File* button to select your flat item table and click *Save*. Alternatively, when the column header names in the CSV file are identical to those in the default flat item table, this can be copied over from the starter content in the same way as the item response table. The CSV file can then be uploaded with the *Restructure* box unchecked. This may improve system performance when collecting a large volume of responses by preserving certain database column types.

### Open the Flow Chart

**Figure 2:**
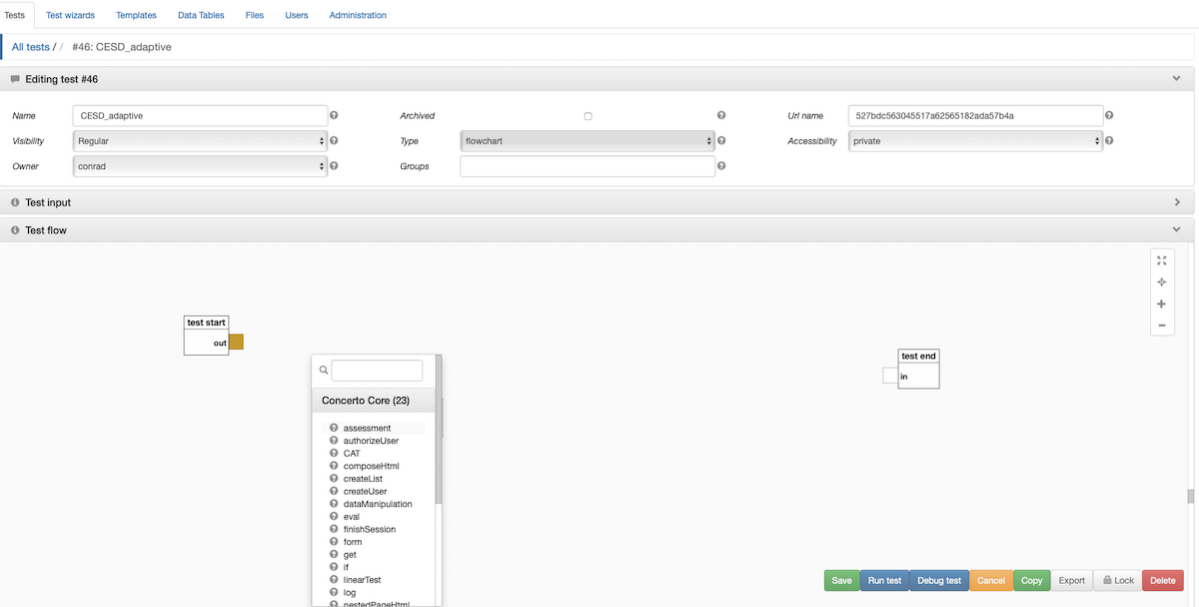
Concerto screenshot: opening the flowchart.

Under the *Tests* tab, click *Edit* next to your assessment. In the *Test flow* window, you will see your assessment displayed as a flow chart. It will have two nodes: *test start* and *test end*, which have a yellow output port and a white input port, respectively. Drag the *test end* node towards the right-hand side of the window to create some empty space between your nodes. Right-click the space between the nodes to create a third node (see Figure 2). Select *assessment*. The assessment node wizard should open automatically, with the *Items* tab preselected.

### Customize Your CAT

Under *Type*, select *Flat Table* from the drop-down menu. Click the *launch setter dialog* icon under *Flat Table*. Select your item table from the *Table* drop-down menu and click *Save*. Select *CAT* from the *Order* drop-down menu.

Under the *Stopping Rules* tab, you can set stopping rules for your CAT. Try setting *Minimum Accuracy* to 0.5. This number represents the SEM that your assessment will achieve before terminating.

Under the *CAT Options* tab, select *GRM* from the *Model* drop-down menu. This relates to the psychometric model parameters which we will use for the CAT. In this example, our item parameters relate to a model known as the graded response model (GRM) [27].

Under the *Responses* tab, click the *launch setter dialog* icon next to *Response Bank*. Select your responses table from the *Table* drop-down menu. Click *Save*. Check the boxes next to *Calculate Theta* and *Calculate SEM*.

Under the *Templates* tab, enter a name for your assessment in the *Title* box (eg, CESD). Click the *launch setter dialog* icon under *Instructions*. Type some instructions for your assessment (eg, “Below is a list of the ways you might have felt or behaved - please tell me how often you have felt this way during the past week”). Click *Save*. Uncheck the *Show Page Info* box. Click *Save*.

### Add the CAT to Your Assessment

Connect the *test start* node to the *assessment* node by dragging the yellow output port on the *test start* node to the white input port on the *assessment* node. Click the red plus sign on the assessment node to create new return ports. Check the boxes next to *theta* and *sem* and click *Save*. This enables the *assessment* node to pass on a person’s score (theta) and the SEM associated with that score.

Right click the empty space between your *assessment* node and *test end* node to create a fourth node. Select *scoring*. Choose *Percentile (normal distribution)* from the *Score Type* drop-down menu. Enter a mean of 0 and a standard deviation of 1. Click *Save*.

On the *scoring* node click the blue plus sign to add an input port. Check the box next to *rawScore* and click *Save*. Click the red plus sign on the *scoring* node to add a return port. Check the box next to *score* and click *Save*. Connect the yellow output port from the *assessment* node to the white input port of the *scoring* node. Connect the *theta* return port to the *rawScore* input port.

### Create a Results Page With Contextual Feedback

Right click the empty space between your *scoring* node and your *test end* node to create a fifth node; you may need to reposition the nodes to make sufficient space. Select *showPage*. Enter a title (eg, CESD), then click the *launch setter dialog* icon under *Content* and copy the following:

Theta is {{theta}}. SEM is {{sem}}.

Your score is higher than {{percent}}% of the general population.

The double braces (curly brackets) specify which values for our feedback page to take from our scoring node. *Click Save*. Clear the *Button Label* and click *Save* again.

Click the blue plus sign on the *showPage* node to create a new input port. Type theta into the text box, noting the lowercase. Click *Add*.

Click the blue plus sign on the *showPage* node to create a second input port. Type sem into the text box, again using lowercase. Click *Add*.

Click the blue plus sign on the *showPage* node to create a third input port. Type percent into the text box, again using lowercase. Click *Add*.

Connect the *sem* return port on the *assessment* node to the *sem* input port on the *showPage* node.

Connect the *theta* return port on the *assessment* node to the *theta* input port on the *showPage* node.

Connect the *score* return port on the *scoring* node to the *percent* input port on the *showPage* node.

**Figure 3:**
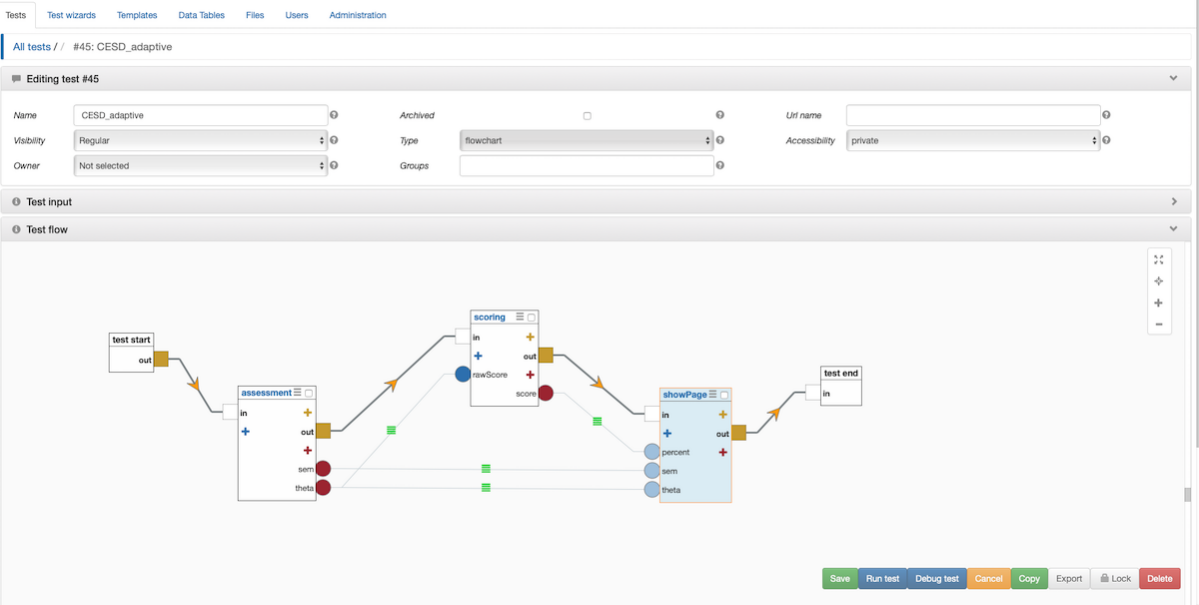
Concerto screenshot: connecting nodes.

Connect the yellow output port on the *scoring* node to the white input port on the *showPage* node. Connect the yellow output port on the *showPage* node to the white input port on the *test end* node (see Figure 3). Click *Save*. You are now ready to run your assessment. Responses will be stored in the CESDResponses table.

## Concerto Case Studies

### Quality of Life Assessment

The World Health Organization Quality of Life (WHOQOL)-100 questionnaire was developed in the 1990s by the WHOQOL group as part of an international collaborative effort to produce a generic, cross-cultural, and widely accepted tool for measuring quality of life. The 100-item questionnaire assesses quality of life across six domains (physical, psychological, social, emotional, independence and spiritual), and is also available as a 26 item (WHOQOL-BREF) version with four domains (physical, psychological, social and environmental) [28].

In 2016, our team calibrated item banks from the 100-item questionnaire using modern test theory and trialed a unidimensional CAT version of the questionnaire using simulated data across four domains (physical, psychological, social and environmental). The CAT version of the questionnaire used 43% and 75% fewer items than the WHOQOL-BREF and WHOQOL-100 questionnaires respectively, at comparable levels of reliability [5]. The WHOQOL CAT, when administered through Concerto, takes a mean of 121 seconds to complete, approximately 10 minutes faster than the WHOQOL-100 [29].

### Analyzing Open-Text Feedback of Doctors’ Performance With Machine Learning

Multisource feedback has become a routine part of UK doctors’ training and appraisal. Often, these feedback assessments contain open-text comments from colleagues about a doctor’s performance [30]. Automating the analysis of these comments could provide real-time, objective insights into both an individual doctor’s performance and the interpersonal dynamics of a team or department. 

In 2017, our team demonstrated the ability for machine learning algorithms to classify open-text feedback from the General Medical Council Colleague Questionnaire (GMC-CQ) into five themes, with human-level accuracy. These themes were innovation, interpersonal skills, popularity, professionalism and respect. Doctors classified as professional, respected or with good interpersonal skills achieved higher GMC-CQ scores than those who were not classified as such [31]. 

Interested readers can freely apply these algorithms to their own open text comments using the Concerto-based platform [32].

### Improving Assessments of Patient Experience With Machine Learning

Patient-reported experience measures (PREMs) that take the form of questionnaires are often limited by a *ceiling effect*. This describes a skew towards positive reporting, which can limit the discriminative ability of a PREM and mask poor service performance [33,34].

We have shown that in the context of UK primary care, spoken feedback from patients can provide more accurate, more detailed, and more actionable insights into the consultation experience than questionnaire results alone. In one study, we found a tendency for patients to rate consultation experiences positively when answering items from the interpersonal skills domain of the national GP Patient Survey, although nearly 60% of respondents who rated their consultation as “good” provided contradictory feedback when interviewed about their experience [35].

Using Concerto, we have developed a patient satisfaction assessment called INSPiRES (Innovative Systems for Patient Reported Experience in Surgery) that combines multiple choice responses with open-text analysis. During the assessment, respondents first select 1 of 12 emotions that describe how they feel about the care they have received. Next, respondents provide an open-text description indicating why they feel that way. Finally, respondents explain which part of their experience led to that feeling (eg, waiting times, cleanliness, care providers) by either selecting a prespecified option or entering free text. The tool is being trialed at the Brigham and Women’s Hospital, Boston, MA, USA, and is expected to provide specific, actionable feedback that will drive service improvement [36]. Although currently used during surgical outpatient clinics, the assessment is also available as a smartphone app that patients can complete from home.

## Discussion

In this article, we have introduced Concerto and demonstrated how to create an advanced, adaptive assessment, for free, with minimal prior experience of CAT or programming. Concerto assessments can incorporate other features, including those that use machine learning, although this is less straightforward at present. The platform has been used internationally to improve the performance of PROMs in research and clinical practice, to classify open-text assessments of health care providers, and to provide meaningful insights into the experience of health care delivery [2,24,31,32,36].

In future, Concerto could be used to develop and deploy advanced clinical decision support systems (CDSSs) that harness the power of CAT and machine learning to assist clinicians in making evidence-based decisions during daily practice. These systems, which can use patient-reported assessments to predict the outcomes of an individual following a health care intervention, are already being trialed to streamline UK GP referrals and support shared decision making in surgery [37]. Existing CDSSs, most notably the NHS Pathways CDSS, which is used by NHS 111 to triage over 14 million telephone calls a year [38], could be trained to automatically interpret spoken word or open-text through natural language processing.

Concerto-based assessments can be deployed on mobile devices as a tool for remote symptom monitoring. Besides the survival advantage this can bring patients with cancer [16], it has quite obvious implications for a broad range of domiciliary disciplines (eg, out-of-hospital palliative care, general practice, and psychiatry).

Patient-reported assessments can transform clinical practice, research, commissioning, and health care management strategies by measuring the impact of an intervention from the patient’s perspective. To deliver the full potential of these assessments, they should be short, accurate, and acceptable to both respondents and those administering the assessment. They should be personalized, ask only the most relevant questions to an individual, and not be limited to multiple-choice responses. Results should be analyzed in real-time and presented to assessment users in an engaging and meaningful way. Where appropriate, data should be easily available for use in secondary analyses including predictive models. These assessments must integrate easily with health care services, including interoperating with electronic health records. Patient data must be stored and processed securely and ethically.

The Concerto platform bridges the implementation gap between the assessments of today and those of tomorrow.

## Abbreviations

CAT: computerized adaptive test
CDSS: clinical decision support system
CES-D: Centre for Epidemiologic Studies Depression Scale
GMC-CQ: General Medical Council colleague questionnaire
GRM: graded response model
INSPiRES: Innovative Systems for Patient Reported Experience in Surgery
NLP: natural language processing
PREM: patient-reported experience measure
PRO: patient reported outcome
PROM: patient-reported outcome measure
PROMIS: Patient-Reported Outcome Measurement Information System
SEM: standard error of measurement
WHOQOL: World Health Organization quality of life
